# Investigating the Effects of Different Sizes of Silicone Rubber Vacuum Extractors during the Course of Delivery on the Fetal Head: A Finite Element Analysis Study

**DOI:** 10.3390/polym14040723

**Published:** 2022-02-14

**Authors:** Chuang-Yen Huang, Kuo-Min Su, Hsueh-Hsing Pan, Fung-Wei Chang, Yu-Ju Lai, Hung-Chih Chang, Yu-Chi Chen, Chi-Kang Lin, Kuo-Chih Su

**Affiliations:** 1 Department of Obstetrics and Gynecology, Tri-Service General Hospital, National Defense Medical Center, Taipei 114, Taiwan; jacky90621@gmail.com (C.-Y.H.); aeolusfield@hotmail.com (K.-M.S.); doc30666@gmail.com (F.-W.C.); yuru220@hotmail.com (Y.-J.L.); 2 School of Nursing, National Defense Medical Center, Taipei 114, Taiwan; pshing2001@mail.ndmctsgh.edu.tw; 3 Department of Nursing, Tri-Service General Hospital, National Defense Medical Center, Taipei 114, Taiwan; 4 Department of Biomedical Engineering, Hungkuang University, Taichung 433, Taiwan; hcchang@sunris.hk.edu.tw (H.-C.C.); cs3401@sunrise.hk.edu.tw (Y.-C.C.); 5 Department of Medical Research, Taichung Veterans General Hospital, Taichung 407, Taiwan; 6 Department of Chemical and Materials Engineering, Tunghai University, Taichung 407, Taiwan

**Keywords:** silicone rubber vacuum extractors, operative delivery, biomechanics, finite element analysis

## Abstract

During certain clinical situations, some parturients require instruments for operative vaginal delivery, and various designs of vacuum extractors may affect the fetal head. To investigate the biomechanical effects of divergent sizes of silicone rubber vacuum extractors, we employed finite element analysis in this study. First, we constructed computer models for different vacuum extractor sizes (diameters: 40 mm, 50 mm, 60 mm, and 70 mm), flat surface, hemispherical ball, and fetal head shape. A hemispherical ball was the main design for the vacuum extractor model, and the material used for the vacuum extractor was silicone rubber. Next, the settings of 1 mm vacuum extractor displacement and vacuum cap pressure of 60 cmHg were applied. The main observation markers of this study were the respective von Mises stresses on the vacuum extractor and skull by the reaction force on the fixed end. The concluded results revealed that vacuum extractors with larger diameters lead to greater reaction force, stress, and strain on fetal heads. Therefore, this study’s biomechanical analytic consequences suggest that clinicians avoid selecting larger vacuum extractors during operative instrumental delivery so that fetal heads will experience less external force, deformation, and resultant complications. It could also provide a practical reference for obstetricians for instrumental vaginal delivery with the vacuum extractor made of silicone rubber.

## 1. Introduction

Most deliveries are normal spontaneous delivery via the natural vaginal pathway. However, 12% of deliveries require instrumental aid under special circumstances for a successful delivery. Such deliveries can be classified as forceps-assisted and vacuum extractor suction deliveries [1]. The American College of Obstetricians and Gynecologists (ACOG) published guidelines on the use of operative vaginal delivery, which included a list of indications for its use such as prolonged second stage of labor, nonreassuring fetal status, elective shortening of the second stage of labor, and maternal exhaustion [2]. In 2017, 3.1% of all deliveries were accomplished by an operative vaginal approach [3]. The most significant disadvantage of using instruments for operative vaginal approach is that there is a higher chance of having a vaginal tear that involves the muscle or wall of the anus or rectum, known as a third- or fourth-degree perineal laceration; the overall risk of complications due to the use of obstetrical forceps is higher than the use of vacuum extractors; thus, the use of vacuum extractors has a lower risk of vaginal tearing and many clinicians employ vacuum extractors to perform operative vaginal deliveries at present [4,5,6,7]. However, uncertain positions, uncertain traction directions, and repeated use of vacuum cups associated with the use of vacuum extractors can lead to cephalohematoma [8], subgaleal and even intracranial hemorrhage [1] of fetus or neonates.

Vacuum extraction accounts for over 80% of operative vaginal deliveries [3]. Vacuum extractors mainly use suction cups with a suctioning capacity of up to 60 cmHg pressure. Vacuum extractors are used to create a suction on the infant’s head thus creating a vacuum between the fetal head and the vacuum cup. Following that, the infant is pulled out of the mother’s vagina for delivery. Currently, suction cups made of different materials are commercially available and comprise mainly of silicone rubber and metal. Based on clinical usage experience, excessive suction force will cause scalp wounds, skull fracture, and intracranial bleeding [7] in the infants. Silicone rubber tends to detach. However, silicone rubber vacuum extractors cause less injury to the infant head compared to the metallic vacuum extractors [9]. The selection of an appropriate vacuum extractor can provide a reliable basis for avoiding obstetric complications such as scalp wound, head hematoma, and even cerebral hemorrhage [10]. In addition, there are different vacuum extractor sizes indicated for different clinical situations. Malmström examined vacuum extractors with different diameters (40–60 mm), and the study results showed that maximum traction increases as the vacuum extractor diameter increases [11]. However, they used metallic vacuum extractors. Moreover, with the progression of 3D printing technology, many medical devices could be made by 3D printing customized manufacture, and hence, soft materials by 3D printing could also be applied for vacuum extractor. In addition to allowing large deformations, materials printed by 3D printing with soft material would be made out into different geometric shapes [12]. Thus, 3D printing would be considered as a very suitable procedural model with various materials and methods for clinical designs and research of vacuum suction extractor for operative vaginal delivery in the future. Therefore, it is very important to understand the influence of the geometry and size design of silicone rubber vacuum extractor during the course of events of labor. Although there are many existing studies on vacuum extractors, there is no such study so far on the biomechanical effects of silicone rubber vacuum extractors of diverse sizes.

As fetal heads are located in the vagina during vaginal delivery, it is difficult to observe delivery in clinical practice. Hence, many researchers employed the finite element analysis simulation to evaluate biomechanical effects during delivery. Finite element analysis is often used as a constructive analytical tool for clinical biomechanics and utilized for mechanical simulated analysis of different geometry structures and materials [13]. Therefore, finite element analysis is an appropriate research method for investigating the biomechanical situation of vacuum extractor with different design as the real clinical course of delivery. Some researchers observed the pressure distribution on the infant’s head during the first stage of labor [14]. When pressure is higher, the force will be higher (the simulation results reveal the quantitative relationship between labor force during delivery and fetal skull molding) [15]. In addition, other researchers observed vaginal tears during delivery in the mother. Appropriate execution of the Viennese manual perineal protection seems to decrease perineal tension and this is unrelated to the size of the fetal head. Therefore, this method seems to be suitable for reducing the risk of perineal injury in all pregnant women [16]. Furthermore, researchers have employed finite element analysis to show that the molding of heads (the molding of the fetal head during vaginal delivery facilitates the labor progress, since it adjusts to the birth canal geometry) can result in a successful delivery and reduce the reaction forces by 17% [17]. Moreover, many researchers have carried out biomechanical analysis for obstetrical forceps. Lapeer et al. assessed the different asymmetrical clamps and found that asymmetry may result in greater force [18]. Researchers also analyzed the angle of obstetrical forceps and the study results showed that a larger curve angle of the forceps blades can decrease the stress and pressure on the neck of the newborn; however, it may lead to rotation toward the posterior side [19]. A previous study also employed finite element analysis to observe the biomechanical effects of vacuum extractors on fetal heads [10]. In addition, finite element analysis has been employed to study the biomechanical effects of vacuum extractors in correct and incorrect positions [20]. An incorrect position may cause damage, including lower anatomical structures such as blood vessels and may cause bleeding in different (depth) scalp layers in fetuses. Therefore, it is not easy to observe the effects of external forces applied by vacuum extractors on the fetal heads in clinical practice. Hence, the use of finite element analysis can simulate obstetrical situations in vitro and effectively study the biomechanical effectively of vacuum extractor suction force on fetal heads during delivery.

According to existing literature, although many studies have performed biomechanical analysis of obstetrical forceps or vacuum extractors during delivery, no study carried out a biomechanical assessment on the sizes of silicone rubber vacuum extractors. Therefore, the primary objective of this study was to use the finite element method to examine the effects of different sizes of silicone rubber vacuum extractors on the fetal heads. We hope that the results of this study can provide a biomechanical basis for clinicians to select the vacuum extractor size for operative vaginal delivery; decrease the incidence of caput succedaneum scalp edema, cephalohematoma, subgaleal, and even intracranial hemorrhage; avoid further harm to the mother; and improve overall treatment quality when instrument-assisted delivery is required for vaginal delivery.

## 2. Materials and Methods

### 2.1. The Simulation Geometry Model

**Figure 1:**
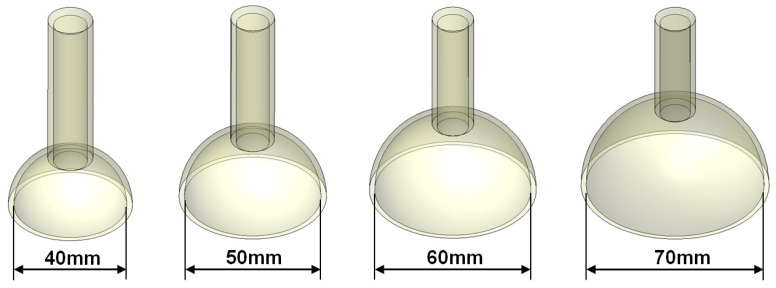
Computer models of 4 vacuum extractors with different sizes.

**Figure 2:**
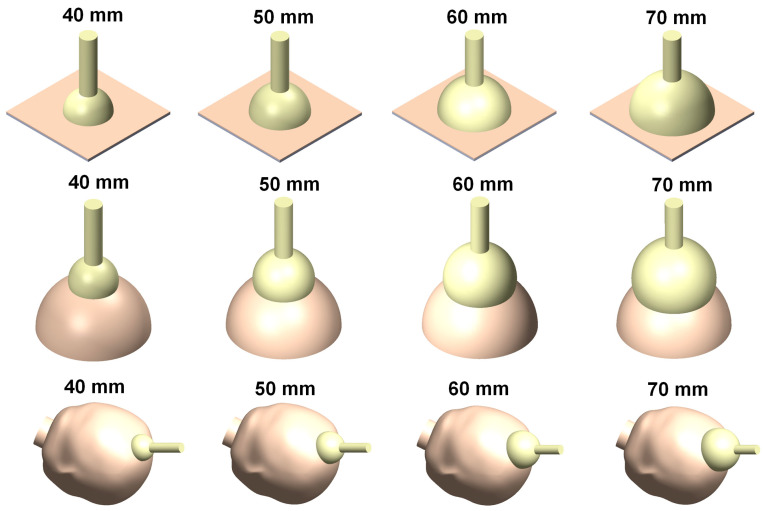
Finite element analysis computer models of vacuum extractors acting on flat surface, hemispherical ball, and fetal head.

In this study, finite element models for four different vacuum extractor sizes were constructed to examine the effects of different silicone rubber vacuum extractor sizes. These vacuum extractor models were mainly hemispherical in design [21], and mainly had diameters of 40 mm, 50 mm, 60 mm, and 70 mm (Figure 1). The three-dimensional (3D) models of these four vacuum extractors were constructed using the 3D computer plotting software Solidworks (Solidworks 2016, Dassault Systemes SolidWorks Corp., Waltham, MA, USA). In order to assess the effects of different vacuum extractor sizes, we separately used vacuum extractors to act on a flat surface, hemispherical ball, and infant head model. The infant head model was mainly based on a previous study, and a geometric appearance of infant’s scalp and skull was constructed based on the anatomical model of neonatal heads [10,19]. For flat surface, hemispherical ball, and fetal head models, two layered structures were used, which were 1 mm-thick scalp and 2 mm-thick skull. The hemispherical ball had a diameter of 10 cm to simulate neonatal head circumference. Therefore, the computer models used in this study were mainly divided into three parts: namely scalp, skull, and vacuum extractors. Furthermore, the CAD software Solidworks was used to combine the scalp, skull, and vacuum extractor (Figure 2). The constructed model was imported into the finite element analysis software ANSYS Workbench (ANSYS Workbench 18.0, ANSYS, Inc., Canonsburg, PA, USA) for finite element analysis.

### 2.2. Loading Conditions and Boundary Conditions

**Figure 3:**
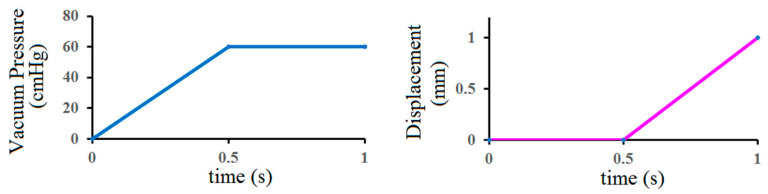
Two load conditions (internal pressure of vacuum extractors and vacuum extractor displacement).

**Figure 4:**
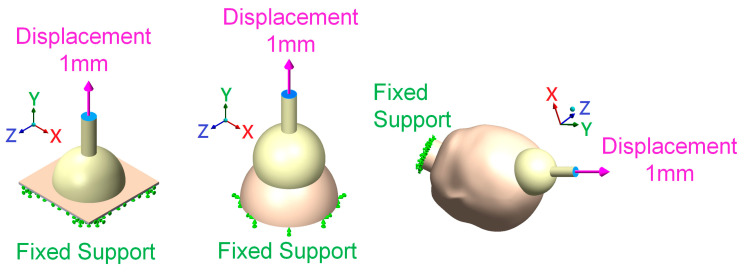
Location of boundary condition and load condition in the finite element analysis computer model.

This study mainly simulated the effects of different silicone rubber vacuum extractor sizes. Therefore, one boundary condition and two different load conditions were provided in this study. Figure 3 shows the two different load conditions used in this study. The first load condition simulated the suctioning by vacuum extractors when the neonatal head was held by obstetrical forceps and the head suction pressure ranged from 0 cmHg to 60 cmHg [21,22,23], which was mainly in the medial side of vacuum extractors. The second load condition was to simulate the outward pull of vacuum extractors. This study mainly used displacement control for Y-directional displacement of the vacuum extractor ends. The displacement ranged from 0 mm to 1 mm (blue region in Figure 4). In addition, the boundary conditions of this study were set, which were mainly toward the bottom of the flat surface, bottom of the hemispherical ball, and the neck of a fixed support (green region in Figure 4). The *x*-axis, *y*-axis, and *z*-axis of this region were set as 0. In terms of contact setting between the fetal head and the vacuum extractor, we also set the contact surfaces between the fetal head and the vacuum extractor as bonded, indicating that the fetal head and the vacuum extractor will not be separated in the process of finite element analysis.

### 2.3. Material Properties of the Model

**Figure 5:**
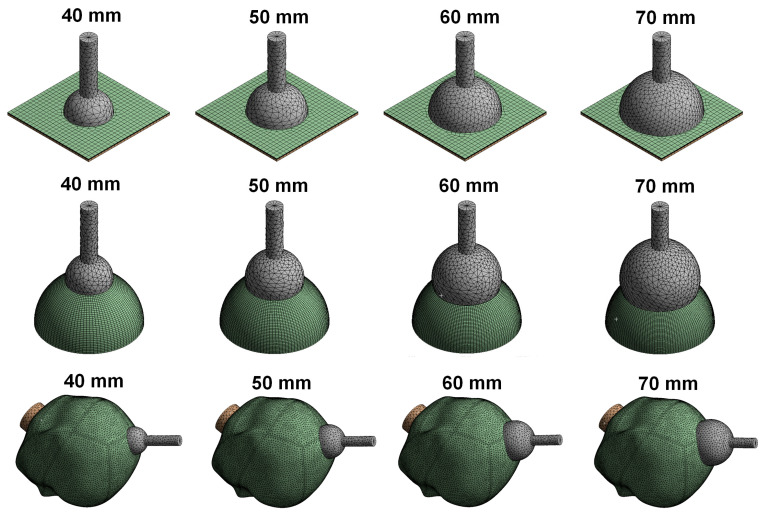
Finite element analysis computer model mesh status of vacuum extractors acting on flat surface, hemispherical ball, and fetal head.

This study model consisted of three parts—namely scalp, skull, and vacuum extractors. The material used for vacuum extractors was mainly silicone rubber. The material property settings used in this study were mainly obtained from other previous studies [24,25]. All materials used were hypothesized to be homogeneous, isotropic, and linearly elastic. Therefore, two independent parameters (Young’s modulus and Poisson’s ratio) were used to express the properties of the materials. Table 1 shows the material property settings used in this study simulation. This study mainly uses finite element analysis software ANSYS Workbench for evaluation. The model meshing elements used in this study are solid 186 (brick with 20 nodes) and Solid 187 (Tetrahedron with 10 nodes) built into the software. For the correctness of finite element analysis, the convergence test is mainly used to achieve the correctness of the solution. The convergence test is mainly based on mesh size control. The mesh sizes of convergence test control are 10 mm, 9 mm, 8 mm, 7 mm, 6 mm, 5 mm, 4 mm, and 3 mm, respectively. The reaction forces were used as the observation index of the convergence test. After the mesh convergence test, the mesh size used in this study is 4 mm. All 12 finite element mesh models reached 5% of the stop criteria of convergence test when mesh size convergence test was used [26]. Therefore, the finite element mesh model used in this study was rational. Figure 5 shows the model mesh used in this study. Table 2. Number of nodes and elements in the computer finite element analysis model used in this study.

After finite element analysis, the main observation markers of this study were the reaction force of the fixed end and von Mises stresses on the vacuum extractor and skull. Where von Mises stress is defined as

(1) $$\sigma_{\mathrm{von}}=\sqrt{\frac{1}{2}[{\left(\sigma_{1}-\sigma_{2}\right)}^{2}+{\left(\sigma_{1}-\sigma_{3}\right)}^{2}+{\left(\sigma_{2}-\sigma_{3}\right)}^{2}]}$$

where σ_1_, σ_2_, and σ_3_ represent the principal stress along the three axes.

In addition, we can evaluate the amount of reaction forces and displacement received by each structure, and the ratio of the two values is called stiffness. As the loading condition in this study is given in the way of giving displacement (1 mm), the reaction forces value of each group can be used to get the stiffness value. These observation markers were used for biomechanical analysis of different sizes of vacuum extractors.

## 3. Results

In this study, finite element analysis was used to obtain the reaction force of various parts and the distribution of other structural stresses. Table 3 mainly shows the reaction force when different sizes of vacuum extractors were used for operative vaginal delivery and acted separately on the flat surface, hemispherical ball, and fetal head. Based on the values shown in Table 3, the higher the diameter of vacuum extractors used, the greater the reaction force experienced by the fixed end. In addition, the differences when same sizes of vacuum extractors act on different objects are not great. In addition, we can evaluate the amount of reaction forces and displacement received by each structure, and the ratio of the two values is called stiffness. As the loading condition in this study is given in the way of giving displacement (1 mm), the reaction forces value of each group can be used to get the stiffness value. Table 4 mainly shows the stiffness value for different groups. The results showed that the trend of stiffness was similar to that of reaction force.

**Figure 6:**
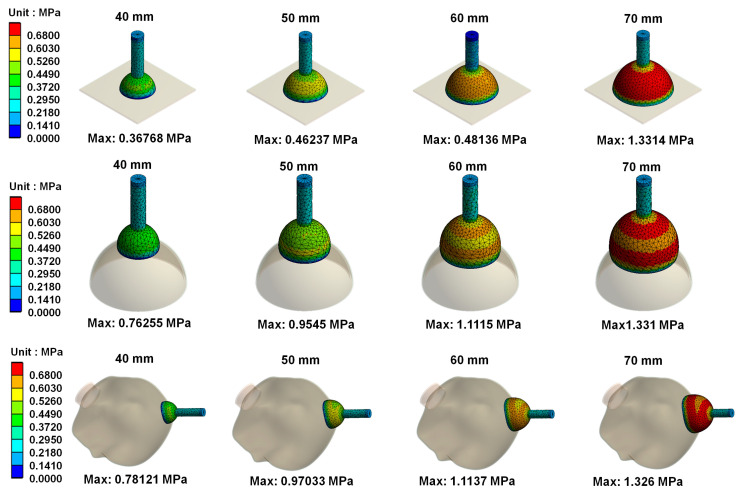
von Mises stress distribution on different vacuum extractors.

**Figure 7:**
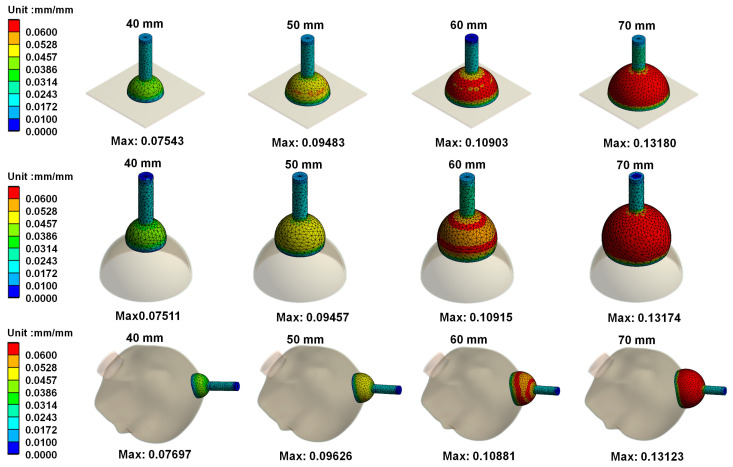
von Mises strain distribution on different vacuum extractors.

Figure 6 mainly shows the on von Mises stress distribution on vacuum extractors when different sizes of vacuum extractors used for operative vaginal delivery acts on different shapes. Results showed that the greater the diameter of vacuum extractors used, the higher the von Mises stress on vacuum extractors. In addition, the differences in von Mises stress on the same size of vacuum extractors when vacuum extractors act on flat surface, hemispherical ball, and fetal head surfaces were not large. Figure 7 shows von Mises strain distribution on different vacuum extractors. The resulting trend of the strain distribution is similar to that of the stress distribution.

**Figure 8:**
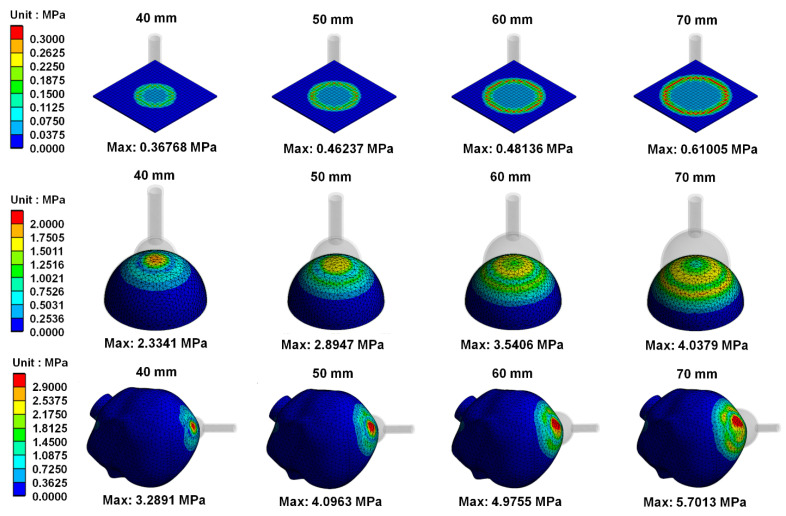
von Mises stress distribution on skull materials when vacuum extractors act on different shapes.

**Figure 9:**
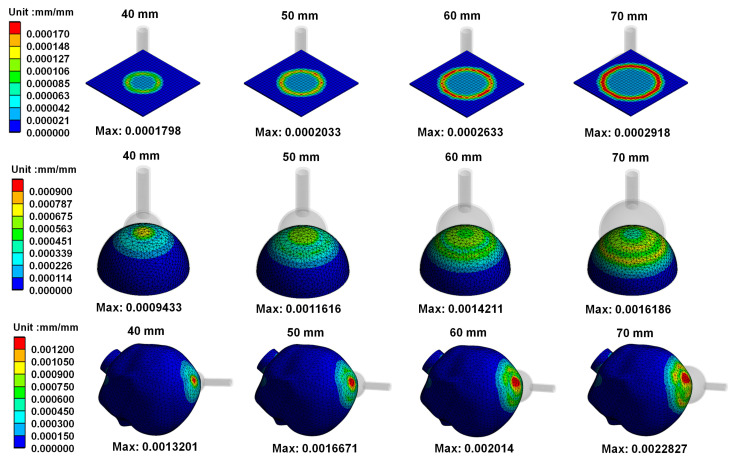
von Mises strain distribution on skull materials when vacuum extractors act on different shapes.

Figure 8 shows the von Mises stress distribution on skull materials when vacuum extractors act on different shapes. Results showed that the greater the diameter of vacuum extractors used, the higher the von Mises stress produced on skull materials. Figure 9 shows von Mises strain distribution on skull materials when vacuum extractors act on different shapes. The results show that when the size of vacuum extractors is larger, the skull structure induces larger strain.

## 4. Discussion

Our study successfully employed finite element analysis to examine the effects of different diameters of vacuum extractors used in operative vaginal deliveries, on flat surfaces, hemispherical balls, and fetal head surfaces. We analyzed the effects of vacuum extractors on flat surface and hemispherical ball to avoid high stress concentration development on the fetal head with the use of vacuum extractors caused by the irregular fetal head geometry. At present, no biomechanical study on the silicone rubber vacuum extractor sizes has been conducted. Therefore, there is no detailed mechanical basis for the effects of different silicone rubber vacuum extractor sizes. The results of this study could provide a biomechanical basis for the selection of vacuum extractor sizes by clinicians. Medical device researchers could improve the design of vacuum extractors based on the results of this study to decrease the negative effects of vacuum extractors on the neonatal head during operative vaginal delivery.

**Figure 10:**
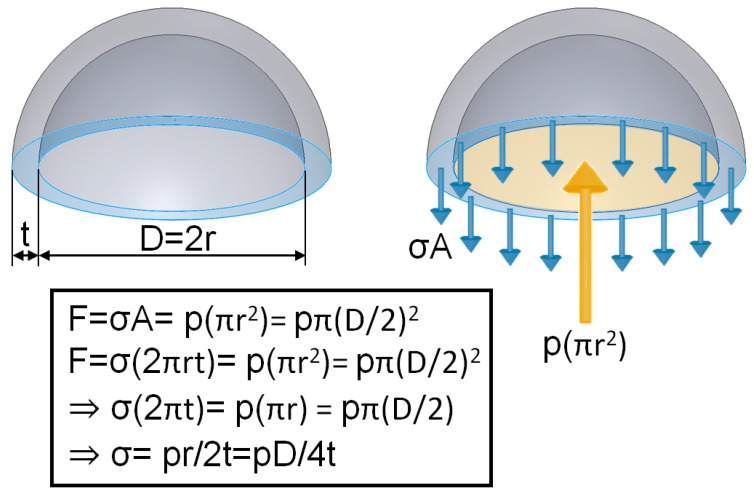
Content in the *Mechanics of Material* textbook was used for explanation. The greater the sphere size (inner radius r), the greater the force [27].

Observation of the reaction force in different groups found that the larger the vacuum extractor size used, the greater the reaction force experienced by the fixed end. The main reason for this could be explained using the content of the Mechanics of Material textbook [27]. The following equations can be used to explain the size of vacuum extractor and reaction force (Figure 10)

(2) $$F=\sigma A=p\left({\pi r}^{2}\right)=p\pi {\left(\frac{D}{2}\right)}^{2}$$

(3) $$F=\sigma \left(2\pi \mathrm{rt}\right)=p\left({\pi r}^{2}\right)=p\pi {\left(\frac{D}{2}\right)}^{2}$$

(4) $$\sigma \left(2\pi t\right)=p\left(\pi r\right)=p\pi \left(\frac{D}{2}\right)$$

(5) $$\sigma =\frac{\mathrm{pr}}{2t}=\frac{\mathrm{pD}}{4t}$$

where σ is stress on vacuum extractors, F is force, A is cross-sectional area, p is vacuum pressure, r is radius of the vacuum extractor, D is diameter of the vacuum extractor, and t is wall thickness of the vacuum extractor.

The external force experienced by vacuum extractors is the product of stress on vacuum extractors (σ) multiplied by the cross-sectional area of the neonatal head (2πrt). Due to the effects of vacuum pressure conversion, the force experienced in the interior of the sphere is the vacuum pressure (p) multiplied by the cross-sectional area of the sphere (πr^2^). Therefore, when vacuum extractors are used on the infant’s head, due to the force equilibrium relationship and force transmission, the force is calculated as mathematical Equation (3) F = σ(2πrt) = p(πr^2^). Hence, when vacuum pressure (p) is a fixed value (the pressure used in this study was 60 cmHg), force will increase when the size of the sphere (inner radius r) increases. Therefore, this study used the flat surfaces, hemispherical balls, and fetal head surfaces for finite element analysis simulation identical trends were observed. In addition, we can also find that when the size of the vacuum extractor is larger, the stiffness is larger, so the resistance to deformation is larger, and the reaction force is larger. These study results were similar to the trends of previous studies even though previous researchers used metallic vacuum extractors and not flexible materials for examination [11]. In addition, previous studies have shown that metal vacuum extractors (358.04–361.37 N) exert more force than non-metal ones (12.229–15.064 N) [10]. When the force of attraction is greater than 135 N, such conditions may increase the risk of sphincter injury in the mother and scalp injury in the baby [28]. Although silicone rubber material was used as the vacuum extractor material in this study, the reaction force values obtained in the study were well below 135 N. Therefore, using silicone rubber as the vacuum extractor material can avoid the risk of scalp injury in the fetal head.

In addition, observation of the stress on vacuum extractors found that the greater the size of vacuum extractors, the greater the stress on vacuum extractors under the same displacement. Based on the mathematical Equation (5) (σ = pr/2t, where σ is the stress experienced by the vacuum extractor and t is the wall thickness of the vacuum extractor), when the same thickness of silicone rubber is used for different vacuum extractors, the stress produced on the vacuum extractor after pulling it for a fixed displacement is directly proportional to the inner radius of the vacuum extractor. Therefore, when the size of vacuum extractor used is large, the high stress produced may destroy soft materials such as silicone rubber. Hence, when using soft materials, we recommend that the vacuum extractor should be slightly thicker to avoid damaging the vacuum extractor during pulling. In addition, observation of the strain on vacuum extractors, the resulting trend of the strain distribution is similar to that of the stress distribution. However, because the materials used in this study are evaluated as linear materials, and because of Hooke’s law σ = Eε (where σ is stress, E is Young’s modulus, ε is strain). Therefore, if we look at the strain results, we see that the strain results tend to be similar to the stress results.

In addition, observation of the stress on simulated skulls found that the greater the size of vacuum extractors, the greater the stress on skull structure. In addition, it was found that when the vacuum extractor acts on a flat surface model, the region near the vacuum extractor has higher stress. When vacuum extractors act on the hemispherical ball and fetal head surfaces, higher stress is produced closer to the surface of the sphere. In this study, the material used for skull simulation was set to be homogeneous, isotropic, and linearly elastic. Therefore, based on Hooke’s law (σ = Eε), when the skull material experiences high stress, there will be greater strain on the skull structure as Young’s modulus is a constant. Therefore, a larger vacuum extractor size may cause greater deformation on the neonatal head.

This finite element analysis study had certain limitations. The material properties of this study were set as linear, homogeneous, and isotropic. The finite element analysis study can be evaluated by using nonlinear material properties. Finite element analysis using nonlinear material properties is challenging (and sometimes unsolvable). However, we certainly hope that nonlinear materials can be used as the material properties of this finite element analysis study. Such a study would be closer to reality. However, the main topic of this study is to evaluate the effects of different sizes of silicone rubber vacuum extractors. In order to evaluate the main factors to be discussed in this study, linear materials are used as analysis materials in this research. It is hoped that this simplification can simplify the research result trend instead of being affected by nonlinear material spines. In terms of reaction force data, we mainly observed the force acting in the region fixed by boundary conditions. This constraint caused the reaction force to be greater than that observed in the actual clinical practice. This is because, during actual delivery, the fetal head moves as and when pulled out. In addition, in the structural model analyzed in this study, all material properties were considered to be homogeneous, isotropic, and linearly elastic, in accordance with most previous biomechanics FEM studies. However, this study focused on the effects of different diameters of vacuum extractors. Therefore, we also simplified the fetal head model by establishing two structures (scalp and skull) in addition to the construction of simple flat surfaces and hemispherical ball models. This simplification allowed us to assess the models we wanted to analyze so that the results could be focused on these different influencing factors that we were concerned about.

This study employed finite element analysis for observation of the effects of different diameters of vacuum extractors for operative vaginal delivery on flat surfaces, hemispherical balls, and fetal heads. The results of this study showed that larger vacuum extractor size may cause greater reaction force, stress, and strain on the fetal head and may also cause the vacuum extractor to experience greater stress. Although there are some differences in the values analyzed in this study and the actual situation, but the trends of the former can represent the actual situation. The design of most medical instruments is mainly based on geometric shapes and materials with adequate clinical effectiveness and safety. In future studies, we will also assess various thicknesses of materials of vacuum extractors for operative vaginal delivery during the process of labor to explore the clinical outcomes in real-world scenarios. In addition, all the analytic results of this study can be used as a constructive reference for designing the appearance of vacuum extractors so that obstetricians and gynecologists could avoid the development of obstetrical complications such as caput succedaneum (scalp edema) of the neonate when using vacuum extractors during delivery and allow all clinicians to successfully use vacuum extractors for operative vaginal delivery.

## 5. Conclusions

This study employed finite element analysis to examine the biomechanics of different diameters of silicone rubber vacuum extractors. The study results found that vacuum extractors with larger diameters resulted in greater reaction force, stress, and strain on the neonatal heads. Therefore, the biomechanical analysis results of this study suggest that clinicians should avoid selecting large vacuum extractors during operative instrumental delivery so that neonatal heads will experience less external force and deformation and prevent caput succedaneum (scalp edema). At the same time, this can prevent damage to silicone rubber vacuum extractors. We hope that these study results help obstetricians and gynecologists to decrease stress on the neonatal heads when using vacuum extractors and obstetrical forceps for delivery. In addition, this study can be used as a biomechanical basis for reference of designing vacuum extractors by medical device design staff. In the future, we can also apply this informative method for biomechanical evaluation of the shape designs (thickness and other parameters) of vacuum extractors and it is full of considerable reference value so that clinical obstetricians would have more conducive and safer equipment for instrumental operative vaginal delivery.

## Figures and Tables

**Table 1:** Material properties setting in this study [24,25].

Material	Young’s Modulus (MPa)	Poisson’s Ratio
Scalp	16.7	0.42
Skull	2500	0.22
Silicone rubber	10.3	0.49

**Table 2:** Number of nodes and elements in the computer finite element analysis model used in this study.

Different Shapes	Mesh	40 mm	50 mm	60 mm	70 mm
Flat surface	Nodes	26,708	39,160	58,294	70,466
	Elements	6127	9253	14,068	17,244
Hemispherical ball	Nodes	38,822	48,834	51,177	66,890
	Elements	10,721	12,542	13,141	17,134
Fetal head	Nodes	130,054	131,198	132,961	134,778
	Elements	64,734	65,270	66,097	66,969

**Table 3:** Reaction forces of various fixed ends.

Different Shapes		40 mm	50 mm	60 mm	70 mm
Flat surface	X Axis	1.0443 × 10^−12^ N	3.2591 × 10^−11^ N	1.2457 × 10^−11^ N	3.3406 × 10^−14^ N
	Y Axis	−14.993 N	−15.881 N	−16.836 N	−18.065 N
	Z Axis	6.9628 × 10^−12^ N	−4.036 × 10^−11^ N	−1.9115 × 10^−11^ N	4.4828 × 10^−12^ N
	Total	14.993 N	15.881 N	16.836 N	18.065 N
Hemispherical ball	X Axis	2.1072 × 10^−10^ N	6.9963 × 10^−11^ N	−1.7853 × 10^−10^ N	−2.5786 × 10^−10^ N
	Y Axis	−14.331 N	−15.369 N	−16.491 N	−17.568 N
	Z Axis	1.1635 × 10^−10^ N	−2.0118 × 10^−13^ N	−7.1072 × 10^−11^ N	6.4573 × 10^−12^ N
	Total	14.331 N	15.369 N	16.491 N	17.568 N
Fetal head	X Axis	6.132 × 10^−5^ N	1.5367 × 10^−4^ N	−4.1151 × 10^−5^ N	7.9199 × 10^−5^ N
	Y Axis	−14.303 N	−15.212 N	−16.206 N	−17.406 N
	Z Axis	−3.5533 × 10^−6^ N	1.4878 × 10^−4^ N	−1.1716 × 10^−5^ N	−1.3267 × 10^−4^ N
	Total	14.303 N	15.212 N	16.206 N	17.406 N

**Table 4:** Stiffness value for different groups.

Stiffness	40 mm	50 mm	60 mm	70 mm
Flat surface	14.993 N/mm	15.881 N/mm	16.836 N/mm	18.065 N/mm
Hemispherical ball	14.331 N/mm	15.369 N/mm	16.491 N/mm	17.568 N/mm
Fetal head	14.303 N/mm	15.212 N/mm	16.206 N/mm	17.406 N/mm

## Data Availability

Not applicable.

## References

[1] Goordyal D., Anderson J., Alazmani A., Culmer P. (2021). An engineering perspective of vacuum assisted delivery devices in obstetrics: A review. Proc. Inst. Mech. Eng. Part H.

[2] American College of Obstetricians and Gynecologists (2000). ACOG Practice Bulletin No 17. Operative Vaginal Delivery.

[3] Martin J.A., Hamilton B.E., Osterman M.J., Driscoll A.K. (2019). Births: Final data for 2018. Natl. Vital. Stat. Rep..

[4] Gurol-Urganci I., Cromwell D.A., Edozien L.C., Mahmood T.A., Adams E.J., Richmond D.H., Templeton A., van der Meulen J.H. (2013). Third-and fourth-degree perineal tears among primiparous women in England between 2000 and 2012: Time trends and risk factors. BJOG-Int. J. Obstet. Gynecol..

[5] Pergialiotis V., Vlachos D., Protopapas A., Pappa K., Vlachos G. (2014). Risk factors for severe perineal lacerations during childbirth. Int. J. Gynecol. Obstet..

[6] Landy H.J., Laughon S.K., Bailit J., Kominiarek M.A., Gonzalez-Quintero V.H., Ramirez M., Haberman S., Hibbard J., Wilkins I., Branch D.W. (2011). Characteristics associated with severe perineal and cervical lacerations during vaginal delivery. Obstet. Gynecol..

[7] Peaceman A.M. (2015). Practice Bulletin No. 154: Operative Vaginal Delivery. Obstet. Gynecol..

[8] O’Mahony F., Hofmeyr G.J., Menon V. (2010). Choice of instruments for assisted vaginal delivery. Cochrane Database Syst. Rev..

[9] D’Antona A., Mottet N., Lenoir P., Toubin C., Bourtembourg A., Ramanah R., Riethmuller D. (2020). Preliminary Study Assessing the Efficiency of a New Singleuse Obstetrical Vacuum Device: Icup2^®^. Arch. Obstet. Gynecol..

[10] Chen Y.-H., Su K.-M., Tsai M.-T., Lin C.-K., Chang C.-C., Su K.-C. (2021). Investigating the Vacuum Extractors of Biomedical Devices of Different Materials and Pressures on the Fetal Head during Delivery. Appl. Sci..

[11] Malmström T. (1957). The vacuum extractor an obstetrical instrument and the parturiometer a tokographic device. Acta Obstet. Gynecol. Scand..

[12] Bodaghi M., Damanpack A., Hu G., Liao W. (2017). Large deformations of soft metamaterials fabricated by 3D printing. Mater. Des..

[13] Baiges J., Codina R., Castanar I., Castillo E. (2020). A finite element reduced-order model based on adaptive mesh refinement and artificial neural networks. Int. J. Numer. Methods Eng..

[14] Lapeer R., Prager R. (2001). Fetal head moulding: Finite element analysis of a fetal skull subjected to uterine pressures during the first stage of labour. J. Biomech..

[15] Pu F., Xu L., Li D., Li S., Sun L., Wang L., Fan Y. (2011). Effect of different labor forces on fetal skull molding. Med. Eng. Phys..

[16] Jansova M., Kalis V., Rusavy Z., Räisänen S., Lobovsky L., Laine K. (2017). Fetal head size and effect of manual perineal protection. PLoS ONE.

[17] Silva M., Oliveira D., Roza T., Brandao S., Parente M., Mascarenhas T., Jorge R.N. (2015). Study on the influence of the fetus head molding on the biomechanical behavior of the pelvic floor muscles, during vaginal delivery. J. Biomech..

[18] Lapeer R., Audinis V., Gerikhanov Z., Dupuis O. (2014). A computer-based simulation of obstetric forceps placement. Med. Image. Comput. Comput. Assist. Interv..

[19] Su K.-M., Yu M.-H., Su H.-Y., Wang Y.-C., Su K.-C. (2016). Investigating biomechanics of different materials and angles of blades of forceps for operative delivery by finite element analysis. J. Mech. Med. Biol..

[20] Lapeer R., Gerikhanov Z., Audinis V. A computer-based simulation of vacuum extraction during childbirth. Proceedings of the SIMULIA Regional User Meeting RUM 2014.

[21] Sullivan C., Hayman R. (2008). Instrumental vaginal delivery. Obstet. Gynaecol. Reprod. Med..

[22] Suwannachat B., Laopaiboon M., Tonmat S., Siriwachirachai T., Teerapong S., Winiyakul N., Thinkhamrop J., Lumbiganon P. (2011). Rapid versus stepwise application of negative pressure in vacuum extraction-assisted vaginal delivery: A multicentre randomised controlled non-inferiority trial. BJOG-Int. J. Obstet. Gynaecol..

[23] Suwannachat B., Lumbiganon P., Laopaiboon M. (2012). Rapid versus stepwise negative pressure application for vacuum extraction assisted vaginal delivery. Cochrane Database Syst. Rev..

[24] Roth S., Raul J.-S., Ludes B., Willinger R. (2007). Finite element analysis of impact and shaking inflicted to a child. Int. J. Legal. Med..

[25] Verbruggen S.W., Loo J.H., Hayat T.T., Hajnal J.V., Rutherford M.A., Phillips A.T., Nowlan N.C. (2016). Modeling the biomechanics of fetal movements. Biomech. Model. Mechanobiol..

[26] Su K.-C., Chen K.-H., Pan C.-C., Lee C.-H. (2021). Biomechanical Evaluation of Cortical Bone Trajectory Fixation with Traditional Pedicle Screw in the Lumbar Spine: A Finite Element Study. Appl. Sci..

[27] Gere G. (2001). Mechanics of Materials.

[28] Vacca A. (1992). Handbook of Vacuum Extraction in Obstetric Practice.

